# Normal Aging Induces Changes in the Brain and Neurodegeneration Progress: Review of the Structural, Biochemical, Metabolic, Cellular, and Molecular Changes

**DOI:** 10.3389/fnagi.2022.931536

**Published:** 2022-06-30

**Authors:** Jiseon Lee, Hee-Jin Kim

**Affiliations:** Department of Neurology, Hanyang University Hospital, Seoul, South Korea

**Keywords:** normal aging, neurodegeneration, microscopic changes, structural changes, cellular changes

## Abstract

Aging is accompanied by many changes in brain and contributes to progressive cognitive decline. In contrast to pathological changes in brain, normal aging brain changes have relatively mild but important changes in structural, biochemical and molecular level. Representatively, aging associated brain changes include atrophy of tissues, alteration in neurotransmitters and damage accumulation in cellular environment. These effects have causative link with age associated changes which ultimately results in cognitive decline. Although several evidences were found in normal aging changes of brain, it is not clearly integrated. Figuring out aging related changes in brain is important as aging is the process that everyone goes through, and comprehensive understanding may help to progress further studies. This review clarifies normal aging brain changes in an asymptotic and comprehensive manner, from a gross level to a microscopic and molecular level, and discusses potential approaches to seek the changes with cognitive decline.

## Introduction

Due to improved economic status and public health care systems, global populations have expanded, and life expectancy has increased. In Korea, the aging population is increasing very fast (Baek et al., 2021). It has been reported that approximately 5% of the global elderly population is affected by dementia. The number of patients with dementia has doubled or tripled within the last few decades (2020). Including dementia and neurodegenerative diseases are associated with brain aging. Brain aging is associated with anatomical and functional deterioration, which can result in neurodegenerative diseases.

Neurons in the brain are post-mitotic cells that require regulation systems that keep the levels of neuronal electrical activity consistently to maintain their neuronal network. However, dysfunction of energy metabolism could jeopardize this system and lead to aging of the brain.

According to previous studies, brain aging shows several hallmarks in other tissues as follows (Mattson and Arumugam, 2018): (1) mitochondrial dysfunction; (2) intracellular accumulation of oxidatively damaged proteins, nucleic acids, and lipids; (3) dysregulated energy metabolism; (4) impaired cellular waste disposal mechanisms; (5) impaired adaptive stress response signaling; (6) compromised DNA repair; (7) aberrant neuronal network activity; (8) dysregulated neuronal Ca^2+^handling; (9) stem cell exhaustion; and (10) inflammation. Chronic positive energy balance accelerates brain aging and leads to neurodegenerative diseases through mitochondrial dysfunction, neurotoxic protein accumulation, and neuro-inflammation (Mattson and Arumugam, 2018).

This review summarizes the structural, biochemical, metabolic, cellular, and molecular changes that occur with normal brain aging (Table 1). In terms of structural changes, both gross and microscopic changes occur with aging. Biochemical and metabolic changes can be categorized into different neurotransmitter groups such as acetylcholine, monoamine, and hormones. Cellular and molecular changes in the brain affect the nucleus and mitochondria, oxidatively damage molecules, lysosome and proteasome function, electrophysiological regulation and neuronal calcium homeostasis.

**TABLE 1:** Summary of the aging process in the brain.

Gross changes	Microscopic changes
**Structural Aging**	
● Volume loss	● Lipofuscin accumulation
● Neurodegeneration in the GM, Demyelination in the WM, Ventricular enlargement	● Neurofibrillary tangles, amyloid plaque formation
● Sulci widening	● Dendritic tree decrease, axon number decreases, demyelination
● Cerebrovascular diseases	

**Acetylcholine**	**Monoamine**	**Neurosteroid**

**Biochemical and Metabolic Aging**		
● Dysfunction in the cholinergic system: memory decline	● Disturbed dopaminergic pathway	● Decreased testosterone disturbs BBB, and invoke inflammation activity
● Nicotinic binding ability lost	● Disturbed serotonergic pathway	● Decreased androgen and estrogen reduce receptor expression and synapse density.
● Nicotinic acetylcholine receptors decrease	● Decreased receptors and binding ability	

**Changes in the nucleus**	**Mitochondrial dysfunction**	**Accumulation of** **oxidatively damaged molecules**

**Cellular and Molecular aging**		
● Alteration in gene expression	● mtDNA damage	● ETC damage
● Decrease in synaptic function	● Disturbed ATP production	● Lipid, protein DNA/RNA damage
● Increase in stress responses	● Apoptosis induced	● Disturb cellular metabolic pathway or homeostasis
● Telomere shortening		

**Impaired lysosome and proteosome function**	**Electrophysiological Changes** **in the Brain by Aging**	**Dysregulation of neuronal calcium homeostasis**

● Autophagy and UPS reduction ● Increase cellular wastes	● AP threshold changes: changes in voltage-gated Na^+^ channel activation prperties and subtypes expression pattern. ● AP amplitude decrease. ● AP axonal conduction rate decrease	● Plasma membrane proteins: - Decreased NMDAR function - Post-translational alterations in receptors - Increased VDCC number ● Changes in Ca^2+^ influx and disturb cellular Ca^2+^homeostasis - Cellular organelles - Mitochondria : Depolarization disturbs electrochemical gradients - ER : Ca^2+^regulating receptors changes. : Changes in calcium-binding protein : Decreased expression and disturbs Ca^2+^buffering

*GM, gray matter; WM, white matter; BBB, brain blood barrier; mtDNA, mitochondrial DNA; ETC, electron transport complex; UPS, ubiquitin proteasome system; AP, action potential; NMDAR, N-methyl-D-aspartate receptor; VDCC, voltage-dependent calcium channel; ER, endoplasmic reticulum; ATP, adenosine triphosphate.*

## Structural Changes

### Gross Changes

The brain undergoes various morphological changes with aging, such as cerebral atrophy, gray and white matter changes (GM and WM, respectively), volume loss, ventricular enlargement, and sulci widening (Blinkouskaya and Weickenmeier, 2021). It is widely known that the volume of the brain and its weight decreases with age at a rate of around 5% per decade after 40 years of age (Peters, 2006). Furthermore, the rate of decline may acutely increases after 70 years old (Peters, 2006). For example, a large cross-sectional study of 2200 participants aged 34–97 years suggests lobar volume loss during aging (DeCarli et al., 2005). The frontal lobe volume decrease about 12% across the cohort (DeCarli et al., 2005). The temporal lobe volume declined about 9% (DeCarli et al., 2005). The occipital and parietal lobes shows no significant age-related volume change (DeCarli et al., 2005). It is known that the volume loss is accompanied by expansion of ventricular volume and other cerebrospinal fluid spaces (Anderton, 2002).

In GM, cerebral atrophy occurs through morphological alterations related to a decrease in the complexity of dendrite arborization (Blinkouskaya and Weickenmeier, 2021). Dendritic shortening, loss of dendritic shortening, and decreased dendritic spines trigger a progressive reduction in synaptic density and synaptic transmission, with major consequences on cognitive decline (Dickstein et al., 2007). In contrast, in WM, the common features of tissue changes are partial loss of myelin, axons, oligodendroglial cells, and mild reactive astrocytic gliosis linked to WM lesions (Schmidt et al., 2011). Furthermore, WM changes are related to arteriolosclerosis of small vessels, resulting in incomplete ischemia and cell death and the emergence of perivascular spaces that interfere with the glymphatic drainage of the brain’s waste products (Nedergaard and Goldman, 2020). During normal aging, the GM volume fraction has been reported to drop from 52.35% in those in their 40s to 50.49% in those in their 80s (Taki et al., 2011). The WM has been reported to decrease from 47.63% in those in their 40s to 40.29% in those in their 80s, whereas the ventricular volume fraction increases from 3.22 to 5.66% (Blinkouskaya and Weickenmeier, 2021). In addition, ventricular enlargement is caused by an increase in the space between folds and loss of gyrification (Blinkouskaya and Weickenmeier, 2021).

With respect to the sulci and gyrus of the cerebral cortex, the grooves and folds or ridges, respectively, become wider and shallower with aging (Jin et al., 2018). Sulci modifications result from shrinkage in the gyri, both nearby and distally. They may affect the global shape of the brain with many regional outcomes (Jin et al., 2018). These integral forces are provoked by the combined changes in the cortical GM and WM, along with other subcortical structures. Sulci morphology differences indicate that the sulci are wider (17.3%) in older people (Jin et al., 2018). In addition, the sulci depth has been reported to be shallower in older participants in the intraparietal sulcus. Moreover, sulci width is generally associated with local brain volumetric differences (Jin et al., 2018).

A sufficient supply of blood and structural and functional blood vessels is important for normal brain function (Sweeney et al., 2018). In the brain, cerebrovascular reactivity is crucially important in maintaining cerebral autoregulation and cerebral blood flow over a large range of arterial pressures (Payne, 2016). However, the aging process changes complex interactions between the brain parenchyma and cerebrovascular system, and these changes have effects on health and functioning that negatively impact cognition. Along with arterial inflammation, arteriosclerosis is one of the earliest measurable changes in vascular function (Anderson, 2006). Arterial stiffening occurs before many negative changes to the rest of the cerebrovascular system, brain, and cognition related to dementia, including Alzheimer’s disease (AD) in the elderly (Suri et al., 2020). Specifically, elastic fibers, collagen, and smooth muscle cells comprise the arterial wall, and these components deteriorate over one’s life due to aging-associated mechanisms (Heinz, 2021). Carotid artery dispensability and compliance start to decrease around 30 years old (Heinz, 2021). However, elastin fibers are not normally generated when they are impaired, whereas collagen, which increases the stiffness, is produced (Heinz, 2021). CBF pulsatility is directly related to carotid pulse pressure and predictive WM hyperintensities (Zimmerman et al., 2021). Systolic blood pressure has been associated with GM volume loss in a cross-sectional study (Schaare et al., 2019), and can result in cerebrovascular injuries, accelerated atrophy, WM abnormalities, and asymptomatic infarcts (Maillard et al., 2012). In addition, cerebral vasculature is associated with cognitive function, as metabolic demand decreases with increasing age and functional adult neurogenesis. Specifically, large-vessel factors, such as atherosclerosis, increase the risk of AD and may play a role in vertebral vessel amyloid deposition (Peters, 2006).

### Microscopic Changes

Microscopically, lipofuscins, neurofibrillary tangles, and senile plaques form and increase with age (Moreno-Garcia et al., 2018). Lipofuscin accumulates in some neurons. Lipofuscin contains peroxidased proteins and lipids and may express the frequent failure of cells to clear these products of peroxidation-induced cell damage (Anderton, 2002). With aging, cellular proteostasis declines and disturbs the degradation of misfolded proteins that are prone to aggregating into aggrosomes (Klaips et al., 2018). Normally, lipofuscin is located in the lysosome, as macroautophagy triggers lysosomes to uptake these aggregates (Moreno-Garcia et al., 2018). However, when macroautophagy pathways are blocked for reasons such as aging, lipofuscin can accumulate in the cytosol (Moreno-Garcia et al., 2018). In normal aged mammalian brains, lipofuscins correlate with a specific senescence pattern that alters the neuronal cytoskeleton and cellular trafficking (Moreno-Garcia et al., 2018). The normal brain aging process involves intraneuronal deposits of lipofuscin and neuromelanin pigment (Moreno-Garcia et al., 2018). In contrast, in neurodegenerative disorders, lipofuscin accumulation increases with age and pathological processes such as neuronal loss or cellular alterations related to oxidative stress, proteasome, lysosomal, and mitochondrial dysfunction, which will be described below (Moreno-Garcia et al., 2018).

In the process of aging, toxic proteins, such as amyloid-beta (Aβ) protein in AD and tau in frontotemporal dementia, are potential contributory factors in cognitive decline (Nikhra, 2017). Normally, amyloid plaques and neurofibrillary tangles are considered hallmarks of AD. However, it is observed during normal aging (Guillozet et al., 2003). Aβ is a protein deposited in the brain in some individuals with aging (Guillozet et al., 2003). In addition, Aβ is higher in some normal individuals with mild cognitive impairment (MCI) than in normal older adults (Guillozet et al., 2003). When neuropsychologically normal, healthy older adults have significant neuropathology at autopsy, and amyloid is common because of the form of amyloid deposition (Rodrigue et al., 2009). The number of tangles located in the cell body of affected neurons is low and restricted to the hippocampus, amygdala, and entorhinal cortex in normal aging (Hof et al., 1996). However, when minimal neurofibrillary tangles are present, they are located in the transentorhinal region of individuals without dementia (Braak et al., 2006). When neurofibrillary changes increase in the brain, the entorhinal cortex is also affected, and cognitive impairment, related to the hippocampus, is triggered with further progress (Braak et al., 2006). Paired helical filaments (PHF) and occasional straight filaments comprise neurofibrillary tangles, and it has been shown that patients with AD have neurons severely affected by PHF, as the normal cytoskeleton of microtubules and neurofilaments totally disappear (Bamburg and Bloom, 2009). Therefore, it is believed that a shortage of functional cytoskeleton probably triggers neuronal loss (Anderton, 2002). The contrast between normal and pathological aging is the presence and distribution of neurofibrillary tangles (Bamburg and Bloom, 2009). The number of tangles in each affected tissue is lower in normal aging than in pathological cases and is limited to the olfactory nucleus, para-hippocampal gyrus, amygdala, and entorhinal cortex (Bamburg and Bloom, 2009). In contrast, in pathological aging, the neurodegenerative way, in other words, neurofibrillary tangles are often found with amyloid plaque in patients with AD (Nikhra, 2017).

Other microscopic changes include morphological alterations in neurons. It has been revealed that neuronal loss during normal aging occurs slightly (no more than 10%) (Morrison and Baxter, 2012). However, morphological changes in neurons, especially dendrites and axons, are involved in cognitive decline and behavioral changes (Dickstein et al., 2013). With increasing age, the dendritic tree underwent regression. Dendritic shafts decrease in number, become shorter and less branched, and have fewer spines (Dickstein et al., 2013). Moreover, not every spine is affected to the same extent, and in the specific area of the cortex, thin spines are lost (Dickstein et al., 2013). These spines have high motility and plasticity and are believed to be related to learning (Dickstein et al., 2013). Axons may have glycogen inclusion, degenerated mitochondria, and accumulations of filaments (Wang et al., 2021). These modifications may lead to degeneration. Regeneration of axons follows degeneration, and these axons enable regeneration in old age (Huebner and Strittmatter, 2009). However, it is delayed and proceeds at a slower rate in the elderly than in younger people. The materials move along the axon by slow transport, and the speed of this transport decreases with aging. The decreased rate of transport is believed to explain the process of degeneration of axons (Huebner and Strittmatter, 2009). Furthermore, the myelin sheaths of nerve fibers and synapses are also affected by aging. The myelin sheath plays a crucial role in the rapid propagation of action potentials by providing insulation to axons (Suminaite et al., 2019). However, they are altered by demyelination, remyelination, and myelin decomposition. Through these changes, myelin sheaths lose conduction velocity, and the degrees are slowed with aging (Peters, 2007). In addition, the number of synapses is reduced, with the rate ranging from 15 to 50% depending on the species and the region of the nervous system (Peters, 2006). This alteration is associated with the regression of the postsynaptic structure and loss of presynaptic structures. Although the number of synapses may decrease, the structure of synapses does not change with old age (Pannese, 2011).

## Biochemical and Metabolic Changes

### Acetylcholine

Aging is accompanied by several biological changes in the brain. The manner in which neurons transmit information through nerve impulses is called the action potential. When an action potential is transmitted to the presynaptic terminal of the synapse, it may trigger the release of neurotransmitters. These neurotransmitters are released by synaptic cleavage and bind to synaptic receptors. This process affects other neurons in an exciting or inhibitory manner, causing other neurons to induce action potentials (Lodish et al., 2000). It revealed that various neurotransmitters and receptors change in several regions of the brain during the aging process. Specifically, major neurotransmitter systems can be classified as cholinergic systems, monoamine systems (catecholamine: norepinephrine and dopamine, and indole amine: serotonin), and others such as amino acids, nitro oxide, and hormones (Nikhra, 2017).

Acetylcholine activates skeletal muscles in the somatic nervous system and may affect internal organs in the autonomic systems (Sam and Bordoni, 2022). In particular, cholinergic pathways control cognitive processes and behaviors such as wakefulness, mood, learning motor function, motivation, short-term memory, and a minor part of reward responses (Nikhra, 2017). According to the cholinergic hypothesis, dysfunction of the cholinergic system plays a role in the memory decline often observed in aging and dementia (Araujo et al., 2005). With normal aging, the nicotinic binding ability is lost. In particular, cholinergic neurons and certain nicotinic acetylcholine receptor (nAChRs) subtypes are specifically reduced or eliminated during normal aging (Rogers et al., 1998). It has been revealed that high-affinity nicotine-binding sites in the entorhinal cortex and presubiculum are largely lost in those over 40 years (Utkin, 2019). Moreover, considering that nAchRs trigger the reactivation of neurogenesis by endogenous neural stem/progenitor cells, it is easy to believe that reduction in nAchRs may affect neurogenesis (Utkin, 2019). In fact, it has been identified that a decreased cholinergic input disturbs proliferation, short-term survival, and differentiation into mature neurons, whereas it promotes apoptosis in the hippocampus in mice (Seib and Martin-Villalba, 2015). Cognitive deficits associated with aging and AD are also associated with cholinergic deficits. Therefore, the maintenance of nicotinic receptors may be important for neuronal survival (Rehman and Masson, 2001).

### Monoamine: Catecholamine (Norepinephrine, Dopamine), Indole Amine (Serotonin)

Norepinephrine and dopamine are the most important neurotransmitters that are associated with aging (Nikhra, 2017). They play important roles in the regulation of synaptic plasticity and neurogenesis in the adult brain (Nikhra, 2017). Serotonin and brain-derived neurotrophic factor levels decrease with aging (Nikhra, 2017). Furthermore, monoamine oxidase increases with age and results in the generation of excess free radicals that exceed the inherent antioxidant process (Nikhra, 2017).

Norepinephrine or epinephrine functions in the central nervous system to regulate sleep patterns and alertness. These charges regulate adrenal glands and fight-or-flight responses (Bennun, 2014). Norepinephrine is associated with cognitive processes and behaviors such as anxiety, arousal, circadian rhythm, cognitive control, and working memory (Nikhra, 2017). They also regulate feeding and energy homeostasis, medullary control of respiration, negative emotional memory, nociception and play a minor role in the reward related system (Nikhra, 2017).

Dopaminergic pathways are related to cognitive processes and behaviors such as wakefulness, aversion, cognitive control, working memory (along with norepinephrine), emotion and mood, motivation, motor function, positive reinforcement, reward, sexual arousal, and the refractory period (Schultz, 2007). In age-related changes in the dopaminergic system in the brain, dopamine synthesis, binding ability, and its receptors decrease. According to positron emission tomography (PET) of the normal aged brain, dopamine synthesis is significantly reduced in the striatum and parastriatal region with aging (Harada et al., 2002). In addition, D1, D2, and D3 dopamine receptors also decreased. Dopamine levels decrease by 10% per decade from early adulthood, and in accordance with this change, cognitive and motor performance decline (Rieckmann et al., 2011). That is, with increasing age, the levels of dopamine decline, and synapses/receptors and binding ability are also reduced in the dopaminergic pathway between the frontal cortex and striatum (Ota et al., 2006). Moreover, dopamine levels are associated with various brain regions, such as the anterior cingulate cortex, frontal cortex, lateral temporal cortex, hippocampus, medial temporal cortex, amygdala, medial thalamus, and lateral thalamus (Kaasinen et al., 2000). These deficiencies cause age-related neurological symptoms such as a decrease in arm swing, increase in rigidity, and cognitive flexibility changes (Nikhra, 2017).

The serotonergic pathway is associated with cognitive processes and behaviors such as wakefulness, body temperature regulation, emotion, and mood, including aggression, feeding and energy homeostasis, and sensory perception (Nikhra, 2017). It affects appetite, sleep, memory, learning, temperature, mood behavior, muscle contraction, and function of the cardiovascular and endocrine system (Nikhra, 2017). The number of serotonin receptors and transporters decreases with increasing age. According to PET studies, the number of S1 receptors in the caudate nucleus, putamen, and frontal cerebral cortex decreases. In the same context, the binding capacity for serotonin transporters in the thalamus and midbrain is also diminished (Kumar and Mann, 2014).

## Neurosteroids

The brain is a steroidogenic organ that retains steroidogenic enzymes and produces neurosteroids (Zwain and Yen, 1999). Neurosteroids are steroids produced from various regions of the brain, mainly in the hippocampus, and they are the endogenous regulator of neuronal excitability (Reddy, 2010). The precursors of neurosteroids are the circulating steroid hormones (Reddy, 2010). Neurosteroids belonging to the sex hormones may have neuroprotective effects against brain aging features, especially Aβ- or tau-related toxicity and oxidative stress (Grimm et al., 2016). For example, with the neurosteroid estrogen receptors widely distributed in the brain, estrogen plays a role as a neuroprotective antioxidant against several toxins, which in turn encourage the production of free radical (Norbury et al., 2003). The brain consumes a high rate of oxygen and consists of the neuronal membrane with a high concentration of polyunsaturated fatty acids, factors that leads to the risks of lipid peroxidation (Norbury et al., 2003). Therefore, the antioxidant activity of the brain is required for its homeostasis (Norbury et al., 2003). Estrogen may play important role in the regulation of mitochondrial function both directly and indirectly, yet this process has not been identified clearly (Zarate et al., 2017). Their effects are important to the central nervous system (CNS) which demands high energy (Zarate et al., 2017). Along with the metabolism regulation, estrogen also affects mitochondria in neuronal tissues during biogenesis, apoptosis, and morphology (Zarate et al., 2017). Further, estrogen protects mitochondria from oxidative damage, which may cause mitochondrial DNA mutations (Zarate et al., 2017).

In the senescence process, the decline of neurosteroids, especially that of testosterone in men and estrogen in women after menopause, may impair neuronal function and cause significant age-associated neurodegenerative diseases (Zarate et al., 2017). Testosterone regulates brain functions including dendritic spine morphology, neurogenesis, and learning and memory (Parks, 2020). When testosterone level declines in the serum and brain, the blood–brain barrier (BBB) integrity and the expression of tight junction proteins are disrupted, which may in turn induce an inflammation activity (Parks, 2020). Androgen, a metabolite of testosterone, also carries neuroprotective function (Parks, 2020). Its depletion, which comes with that of testosterone, may stretch into the decline of CA1 spine synapse density (Zarate et al., 2017). Again when age, the local synthesis of estrogen is declined due to the reduced expression of aromatase, the enzyme that synthesizes estrogen utilizing androgen (Li et al., 2014). Additionally, the expression of estrogen receptors in the brain drops during aging, successively causing detrimental impacts on memory and learning by retarding the signaling pathways in the brain areas where estrogen receptors are mediated, that is hippocampus and prefrontal cortex (Zarate et al., 2017). The reduction of synapse number and spine density in the CA1 area of the hippocampus is observed in multiple animal models, especially those with rats, when their ovarian hormones decreased either naturally or artificially (Zarate et al., 2017). Estrogen’s effects on the hippocampus are also shown to be less responsive for older models than younger ones, for there is a significant gap in the number of estrogen receptors between the two groups (Adams et al., 2001).

Sex hormones, or neurosteroids, interact with the insulin receptor, which regulates glucose and carbohydrate metabolism (Baquer et al., 2009). Glucose being the main energy source of the brain, approximately 20% of the total metabolized glucose in the body at a rest state is consumed by it (Mergenthaler et al., 2013). Therefore, disturbed glucose metabolism can lead to various brain malfunction (Mergenthaler et al., 2013). Considering that peptides and steroid hormones control the influx of glucose in cells, the decrease in neurosteroids during aging may pervert glucose homeostasis of the brain (Baquer et al., 2009).

## Cellular and Molecular Aging in the Brain (Figure 1)

### Changes in the Nucleus

Associated with aging, a number of microarray studies have been implemented to identify genome-wide changes in gene expression, particularly in the brain (Yankner et al., 2008). Specific biological pathways change due to the aging process, as opposed to the genome-wide dysregulation of transcription (Yankner et al., 2008). In addition, the expressed genes triggered by the induction of stress are related to aging (Yankner et al., 2008). In a study that conducted transcriptional profiling of the aging human frontal cortex in 30 individuals from 26 to 106 years of age, approximately 4% of the genes expressed in the brain were found to be age-regulated (Lu et al., 2004). Specifically, age-associated changes in gene expression become obvious in middle age and are most clear after 70 years of age (Yankner et al., 2008). Synaptic function-related genes, which mediate memory and learning, were significantly downregulated (Yankner et al., 2008). These genes include glutamate receptor subunits, synaptic and vesicle proteins, and members of major signal transduction systems that mediate long-term potentiation (LTP) (Yankner et al., 2008). For example, synaptic calcium signaling systems are probably affected by decreased expression of calmodulins 1 and 3, several Ca^2+^/calmodulin-dependent protein kinases (CAM kinases), and multiple protein kinase C isoforms (Fraser et al., 2005). Other categories of age-reduced genes are vesicle-mediated protein transport and mitochondrial function (Fraser et al., 2005). In addition, genes related to stress responses, such as antioxidant defense, DNA repair, and immune function, represent the largest category and have been found in several different cortical areas of the aging human brain (Fraser et al., 2005). Gene expression alterations affect the susceptibility of the aging brain to neurodegenerative disorders (Yankner et al., 2008). For instance, a microarray study of AD indicated that a substantial number of expressions are related to pathological markers and cognitive test scores (Blalock et al., 2004). Specifically, it is upregulated in signaling and tumor suppressor genes and downregulated in protein folding, metabolism, and energy-related genes (Blalock et al., 2004). Therefore, neurodegeneration and cognitive decline may be involved in specific alterations in gene expression (Yankner et al., 2008).

**FIGURE 1:**
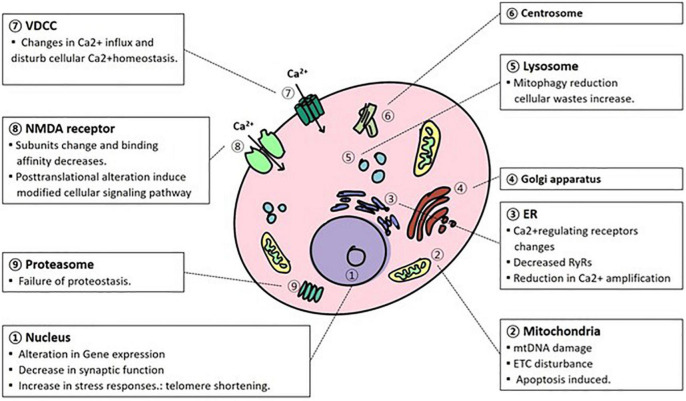
Cellular organism and molecular changes due to aging. In the process of normal aging, various alterations occur in the cellular organelles in a degenerative way. mtDNA, mitochondrial DNA; ETC, electron transport complex; NMDAR, N-methyl-D-aspartate receptor; VDCC, voltage-dependent calcium channel; ER, endoplasmic reticulum.

One of the primary hallmarks is telomere shortening (Lopez-Otin et al., 2013). According to the telomere theory of aging and cellular senescence, cells have a definite number of divisions and define when replication is suitable (Olovnikov, 1996). Telomeres, which have a role in the biological clock, have thousands of tandem DNA repeats, TTAGGG at the end of each linear chromosome (Zia et al., 2021). Telomeres are important because they are engaged in genome maintenance and promote stability through replication procedures, preventing chromosomal fusion and unnecessary recombination (Muraki et al., 2012). Telomerase is a ribonucleoprotein enzyme consisting of two essential subunits: telomerase reverse transcriptase protein (TERT) and telomerase RNA (TER) (Nandakumar and Cech, 2013). It reverses telomere shortening like stem cells (Nandakumar and Cech, 2013). Telomere shortening and its role in the healthy aging process of the brain and neuronal senescence at the cellular level have not been explained (Takubo et al., 2010).

Age-related alterations in neurons are not fully understood (Zia et al., 2021). As neurons are post-mitotic, cell division, the main factor for telomere shortening, has been regarded as absent in neurons when they reach terminal replication (Zia et al., 2021). However, this view has been opposed by the investigation of DNA content variants, describing cell cycle activity in approximately 10–20% of post-mitotic neurons in the cortex of healthy aging brains and AD (Mosch et al., 2007). Telomerase activity restores various features of aging in somatic cells, such as senescence. However, in some somatic tissues, including the central nervous system, the telomerase enzyme has downregulated transcript levels and activity, which are related to protein levels (Wright et al., 1996). In particular, telomerase function in post-mitotic cells, such as neurons, is not related to telomere elongation but is involved in cell survival-promoting function (Zia et al., 2021). Furthermore, hippocampal TERT plays a role in modulating mood behaviors by controlling the proliferation of neural progenitor cells (NPCs) and is needed for spatial memory formation (Rolyan et al., 2011). In this respect, hippocampal-dependent learning and memory functions and neurogenesis in the hippocampus are downregulated in telomerase-knock mice (Rolyan et al., 2011). In addition, when human NPCs are cultured, it is shown that NPCs have a restricted amount of cell divisions and undergo senescence (Wright et al., 2006). Telomere erosion occurs in the aging brain in a cell cycle-independent and dependent way (Zia et al., 2021). In addition, one controversial study reported that leukocyte telomere length is associated with structural changes to the brain and impaired cognitive capacity during aging (Zia et al., 2021).

### Mitochondrial Dysfunction

Mitochondria are intracellular organelles known for producing cellular energy substances (ATP, adenosine triphosphate) and managing cell functions by controlling secondary messengers in cell signaling mechanisms (Calcium ion, Ca^2+^) and reactive oxygen species (ROS) (Misrani et al., 2021). Mitochondria drive the derivation and storage of energy using the respiratory chain *via* oxidative phosphorylation. A single neuron contains hundreds or thousands of mitochondria (Rango and Bresolin, 2018). However, abnormal ROS and calcium ion levels induce mitochondrial damage, which triggers mitochondrial dysfunction. Specifically, mitochondrial damage disturbs mitochondrial DNA (mtDNA) maintenance and ATP production and promotes apoptosis (Ureshino et al., 2014).

The aging process is accompanied by mitochondrial alterations, and key reported features are somatic point mutations and large deletions in mitochondrial mtDNA (Cunnane et al., 2020). These mtDNA mutations alter the transcription of electron transfer complex proteins, which are used in the mitochondrial respiratory system. This alteration has been shown to be accountable for mitochondrial dysfunction, especially in interfering with ATP production and increasing ROS generation (Ureshino et al., 2014). Moreover, mtDNA mutations affect mitochondrial morphology. mtDNA integrity is maintained by mitochondrial dynamics, fusion, and fission (Ureshino et al., 2014). The mtDNA heteroplasmy, a mixture of mutated and normal mtDNA, is formed during mitochondrial dynamics and is controlled by proteins such as mitofusins (Mfs1 and Mfs2), mitochondrial dynamin-like GTPase (Opa1: fusion), and dynamin-related protein 1 (Drp1: fission) (Ureshino et al., 2014). Prevalent fission triggers respiratory impairment and an increase in ROS levels, whereas excess fusion can hinder mitochondrial autophagy induced by large and defective mitochondria (Ureshino et al., 2014).

From the perspective of ATP production, since neuronal cells are highly energy-consuming, mitochondrial dysfunction is considered a serious factor in neuronal disease (Cunnane et al., 2020). In terms of brain energy metabolism, ATP is the main source of the cellular system (Cunnane et al., 2020). Specifically, ATP is consumed by the cell membrane pumps Na^+^/K^+^-ATPase and Ca^2+^ATPase, which regulate ion gradients during neuronal signaling (Cunnane et al., 2020). ATP is also used in the arrangement of neuronal cell organelles (Cunnane et al., 2020). When axons transport mitochondria, RNA, proteins, vesicles, and other cargo to the presynaptic terminal, the process is controlled by calcium, motor protein, and microtubules, ATP is required (Cunnane et al., 2020). Additionally, neuronal networks related to fast-spiking interneurons demand high metabolic energy, and this process is supported by mitochondrial oxidative phosphorylation (OXPHOS) (Cunnane et al., 2020). However, at the cellular level, mitochondrial dysfunction, specifically mutated mtDNA, occurs, leading to a decrease in ATP production generated by OXPHOS, beta-oxidation, and the Tricarboxylic acid cycle(TCA) cycle (Rango and Bresolin, 2018). In particular, AD has features of mitochondrial dysfunction with a lower uptake of glucose and a decrease in the TCA cycle (Rango and Bresolin, 2018). Many PET studies have shown that MCI is related to a 10–12% deficit in glucose uptake (Rango and Bresolin, 2018). Furthermore, this deficit becomes more widespread with the advent of AD and worsens during its progression (Rango and Bresolin, 2018).

Furthermore, mitochondrial dysfunction induces apoptosis and programmed cell death (Bhatia and Sharma, 2021). Mitochondria play a role in the intrinsic apoptotic pathway (Bhatia and Sharma, 2021). The intrinsic apoptotic pathway is the prevalent mechanism of neuronal death. This mechanism involves increased production of ROS, cytochrome c release, which mediates the TCA cycle in mitochondria, ATP depletion, and caspase 9 and 3 activation (Erekat, 2018). Probable triggers of apoptosis include the interaction with α-synuclein (Erekat, 2018). α-Synuclein is abundant in the central nervous system and is a major factor in Lewy bodies, a pathological indicator of PD (Erekat, 2018). Aggregation of α-synuclein in dopaminergic neurons decreases the activity of mitochondrial complex I and increases ROS (Erekat, 2018). ROS and the release of cytochrome c into the cytosol evoke mitochondria-mediated apoptosis as mitochondrial dysfunction occurs (Erekat, 2018). Another factor that causes apoptosis is dopamine metabolism (Erekat, 2018). Dopamine blocks mitochondrial complex I, which leads to mitochondrial dysfunction, and produces anti- and pro-apoptotic factors (Erekat, 2018). Dopamine metabolism is also accompanied by the generation of ROS and dysfunction of the mitochondrial complex I (Erekat, 2018). Damage to the activity of mitochondrial complex I has been suggested to increase the vulnerability of dopaminergic neurons to neuronal disorders by downgrading the threshold for activation of the intrinsic apoptotic pathway (Erekat, 2018). Although it is not clear whether apoptosis induces neurodegeneration in AD, it is certain that several molecules of this pathway are triggered in the AD brain (Bhatia and Sharma, 2021). For instance, caspase 3, an executive caspase that regulates apoptosis, is cleaved and activated in AD and is linked to tau cleavage and neurofibrillary tangle (NFTs) formation (Bhatia and Sharma, 2021).

### Accumulation of Oxidatively Damaged Molecules

Cellular ROS are generally produced by both exogenous and endogenous sources. Exogenous sources of ROS production are ultraviolet (UV) radiation, ionizing radiation, and drugs that mediate their mechanism through ROS production (Martinez et al., 2010). Additionally, environmental toxins and chemicals are responsible for ROS, which are metabolism by-products (Martinez et al., 2010). In the case of endogenous sources, mitochondrial and non-mitochondrial ROS-generating enzyme produces ROS (Martinez et al., 2010). Non-mitochondrial ROS enzymes include nicotinamide adenine dinucleotide (NADH) phosphate oxidase (Nox), xanthine oxidase, cytochrome P450 from the endoplasmic reticulum, and flavin oxidases from peroxisomes (Martinez et al., 2010).

However, most cellular ROS (90%) are generated through mitochondrial ATP production by OXPHOS (Warraich et al., 2020). While the mitochondrial electron transfer chain system (ETC) operates during OXPHOS and produces ATP, complexes I and III of the ETC mainly generate ROS (Warraich et al., 2020). If this damage is not compensated by the antioxidant process, elevated generation of ROS triggers neuronal damage (Bhatia and Sharma, 2021). Antioxidant systems protect against ROS and consist of antioxidant enzymes such as glutathione peroxidase (GPX), non-enzymatic antioxidant factors, superoxide dismutase (SOD), and catalase (Bhatia and Sharma, 2021). During aging, it has been reported that antioxidant capacity decreases (Bhatia and Sharma, 2021).

Molecules damaged by oxidative stress are present in lipids, proteins, DNA, and RNA. Lipid peroxidation (LPO) is triggered by lipids that are attacked by ROS through a free-radical chain mechanism to generate LPO products (Bhatia and Sharma, 2021). In particular, 4-hydroxy-2,3-non-enal (HNE), a common cytotoxic product of LPO, damages neurons and functions of membrane proteins, such as the neuronal glucose transporter GLUT (Bhatia and Sharma, 2021).

In terms of damaged proteins, those vulnerable to oxidative stress can be classified into several groups: metabolic pathways responsible for glycolysis and metabolism, energy metabolism, mitochondrial proteins, cytoskeleton, chaperones, and members of the ubiquitin-proteasome system (UPS) (Martinez et al., 2010). Protein oxidative damage may result in abnormalities in the nervous system, such as abnormal glycolysis and energy metabolism, abnormal protein folding and oxidative stress responses, abnormalities in the cytoskeleton, and damaged protein degradation (Martinez et al., 2010). For example, a number of proteins are sensitive to oxidative stress, such as the chaperone and ubiquitin-proteasome systems, and this damage results in abnormal neuronal function in AD (Martinez et al., 2010). Therefore, it is appropriate to think that some of the metabolic disturbances monitored at the advent of degenerative processes are engaged with oxidative damage of selected proteins other than neuron loss (Martinez et al., 2010).

In the case of mtDNA damaged by oxidative stress, as mentioned above, most ROS are generated from mitochondria, and impaired mtDNA is the common oxidative damaged molecule (Nissanka and Moraes, 2018). Whether mitochondrial ROS affects mtDNA mutations has not been confirmed, but mtDNA changes are more likely to increase ROS beyond normal concentrations, causing neuronal damage (Nissanka and Moraes, 2018). According to the mitochondrial theory of aging proposed by Harman in the 1970s, somatic mtDNA mutations injure OXPHOS complexes, resulting in ROS production (complex I and III) (Pinto and Moraes, 2015). Subsequently, these ROS impair proteins, lipids, and DNA, including mtDNA, and again, these impaired molecules produce new ROS and damage normal molecules. Through this process, a vicious cycle is formed (Pinto and Moraes, 2015).

From the perspective of cellular DNA, oxidative stress can induce DNA double-strand breaks, DNA/protein or DNA/DNA cross-linking, and base modification (Bhatia and Sharma, 2021). DNA bases are sensitive to oxidative damage such as hydroxylation, nitration, and protein carbonylation (Bhatia and Sharma, 2021). Similarly, RNA is commonly single-stranded and is a target of oxidative damage/modification, similar to DNA. Generally, oxidative damage to DNA/RNA is increased in AD (Bhatia and Sharma, 2021). Specifically, 8-hydroxy-2-deoxyguanosine and 8-hydroxyguanosine levels, which indicate DNA and RNA oxidation, are elevated, and these markers are localized in Aβ plaques and NFTs (Bhatia and Sharma, 2021). In addition, the levels of oxidized rRNA or mRNA are also increased in AD (Ding et al., 2005; Bhatia and Sharma, 2021).

### Impaired Lysosome and Proteasome Function

The features of neuronal cells are retained in cellular machinery for protein synthesis and degradation (Tai and Schuman, 2008). In contrast to other cells, neuronal cells have a unique morphology, which is a specific area of presynaptic neurotransmitter release, postsynaptic receptor activation, and plasticity of synapses related to alterations in the synaptic proteome (Tai and Schuman, 2008). Since molecular machinery proteins play a role in mediating signal transduction, protein synthesis and degradation are important for maintaining the plasticity and memory of neuronal cells (Tai and Schuman, 2008). In eukaryotic cells, there are two major degradation systems that provide cell recycling of cellular components, ranging from soluble proteins to intracellular organelles: autophagy, which plays a role in the degradation of long-lived, insoluble or accumulated proteins and cellular organelles, and UPS, which usually degrades the most soluble, short-lived protein. Although they have different mechanisms of action, both play important roles in regulating cell homeostasis (Kocaturk and Gozuacik, 2018).

Cells can digest cytosolic components *via* autophagy lysosomal degradation (Ureshino et al., 2014). In addition to clearing the cytosol for macromolecules and impaired organelles, the autophagy process provides cells with amino acids and energy by recycling, which is energy efficient (Ureshino et al., 2014). Since autophagy plays an important role in the production of long-lived proteins and the elimination of damaged organelles and cellular debris, it is considered a part of the antiaging process (Bergamini, 2006). However, lipofuscins, which are aggregates of insoluble particles formed during the aging process in post-mitotic cells, accumulate in autophagosomes and hinder the autophagic system, which is a critical protective mechanism in the cell (Ureshino et al., 2014). Impairment of the autophagic system, which is induced by aging, causes excessive ROS production from the mitochondrial respiratory system, interrupts recycling, and increases oxidative stress (Ureshino et al., 2014). For example, mitophagy reduction (mitochondrial autophagy) is related to aging-induced diseases such as Parkinson’s disease (PD) (Foltynie et al., 2002). Mitophagy reduction disturbs the removal of Lewy bodies and the aggregation of filamentous intracytoplasmic inclusions, which could trigger brain disorders, such as AD or PD (Ureshino et al., 2014). Another neurodegenerative disease related to autophagy reduction involves genetic alterations and protein expression modification (Ureshino et al., 2014). For instance, changes in PTEN-induced novel kinase 1(PINK1) and parkin, which are related to mitophagy, correspond to 5% of PD (Ureshino et al., 2014). Normally, when mitochondria are impaired, PINK1 aggregates in the outer membrane of mitochondria, parkin is recruited, and mitochondria start to degrade (Ureshino et al., 2014). However, if the PINK1-parkin pathway is dysfunctional due to aging, the homeostatic process conducted by the mitochondria is disturbed (Quinn et al., 2020).

The other perspective of protein degradation is the proteasome (Tai and Schuman, 2008). Proteins that are fated for degradation are tagged with ubiquitin by UPS (Tai and Schuman, 2008). The UPS targets intracellular, soluble, and transmembrane proteins that are extracted from the membrane into the cytosol (Tai and Schuman, 2008). The UPS plays a significant role in the regulation of memory and neurotransmitter release (Tai and Schuman, 2008). The balance between protein synthesis and degradation is responsible for long-term plasticity and memory (Tai and Schuman, 2008). In addition, synaptic transmission at the pre- and postsynaptic terminals is controlled by UPS (Tai and Schuman, 2008). For example, in hippocampal neurons at presynapse, the frequency of miniature excitatory postsynaptic currents increases when the proteasome is inhibited (Yao et al., 2007). In animal models, transgenic animals lacking SCRAPPER showed a large increase in excitatory postsynaptic currents (Yao et al., 2007). Considering that SCRAPPER is an E3 enzyme located in the presynaptic membrane and regulates vesicle release, the UPS at the presynaptic membrane plays an important role in controlling the size of the vesicle pool and vesicle release (Yao et al., 2007). Furthermore, the UPS is responsible for the abundance of proteins that regulate postsynaptic responses, including ionotropic glutamate receptors and proteins related to postsynaptic density (PSD) (Tai and Schuman, 2008). Chronic inhibition of action potentials, such as tetrodotoxin, or of inhibitory neurotransmission, bicuculline, changes PSD proteins (Ehlers, 2003; Tai and Schuman, 2008). Interestingly, these changes are blocked by proteasome inhibitors, which indicates the significance of proteolysis in restructuring the synapse corresponding to changes in neural activity (Tai and Schuman, 2008). In the process of aging, cellular proteostasis declines, misfolds, and damaged proteins aggregate (Saez and Vilchez, 2014). These failures in proteostasis are involved in the stabilization of correctly folded proteins and protein clearance systems (Saez and Vilchez, 2014).

### Electrophysiological Changes in the Brain by Aging

Electrolytes, located in extracellular and intracellular fluid, form electrical currents in the body (Terry, 1994). These ions exercise a crucial role in maintaining homeostasis (Terry, 1994). Along with Ca^2+^, the movement of ions through ionic channels engages in the action potential (AP), the process which is essential for neuronal signals (Strickland et al., 2019). In neurons, ions move rapidly through a whole course of depolarization, repolarization, and signal propagation, producing AP (Levitan et al., 2002). Such ionic channel actions are categorized by which ions participate and how the ion channel gating permeation works (Sather et al., 1994). The voltage-gated ion (Na^+^, K^+^, Cl^–^, and Ca^2+^) channels and ligand-gated ion channels are two examples (Strickland et al., 2019). When depolarization is activated, ions cross the membrane through the channels that match their electrochemical gradients (Strickland et al., 2019). Ion channels in membranes trigger nerve impulses and synaptic transmission (Strickland et al., 2019).

The intrinsic electrophysiological properties change with age (Rizzo et al., 2014). Normally, AP has three main stages: depolarization, repolarization, and hyperpolarization (Grider et al., 2019). Depolarization occurs when the influx of Na^+^ exceeds the AP threshold and the voltage-gated Na^+^ channels are open (Grider et al., 2019). Repolarization happens with the closing of Na^+^ channels and the opening of K^+^ channels (Grider et al., 2019). Hyperpolarization is when excessive K^+^ is accumulated and moves outside the cell through the opened K^+^ channels (Grider et al., 2019). Nonetheless, recent studies show that the AP threshold of the hippocampal CA1 pyramidal cell is higher for aged rats (Matthews et al., 2009). Similarly, even though it has not been fully proven, this age-based variation in the AP threshold may also be applied to humans, affecting the voltage-gated Na^+^ channel activation properties and channel subtype expression patterns (Randall et al., 2012). This variation in the AP threshold may disturb the excitability of neurons and repress neuronal activities by lowering the transmissive function of neurons (Rizzo et al., 2014). Such impairment in the brain might have a correlation with cognitive decline during senescence (Rizzo et al., 2014).

Action potential amplitude takes up a critical role in evoking Ca^2+^ currents and regulating the neurotransmitter release through axon terminals (Rizzo et al., 2014). Some studies show AP amplitude decreases in primates while they age (Rizzo et al., 2014). This phenomenon can be explained by either a reduction of Na^+^ channels or an increase in K^+^ channels (Rizzo et al., 2014). Age-based changes in AP amplitude could be a result of the altered expression of voltage-gated Na^+^ channel subunits, reduced expression of Na^+^, or altered expression of K^+^ channel which involves the K^+^ currents (Rizzo et al., 2014). Additionally, several studies reveal that AP axonal conduction velocity decreases alongside the aging process (Aston-Jones et al., 1985). The cause of such a decrease might be demyelination, the process that induces an ion leakage and reduces the efficiency of transduction (Coggan et al., 2010).

### Dysregulation of Neuronal Calcium Homeostasis

Calcium (Ca^2+^) is a secondary messenger in the cell signaling mechanism (Gleichmann and Mattson, 2011). The optimal concentration of Ca^2+^ remained constant in the cell and extracellular space at the expense of energy (Gleichmann and Mattson, 2011). Through this process, cells promote Ca^2+^-induced signaling pathways and inhibit Ca^2+^ driven excitotoxicity (Chandran et al., 2019). Ca^2+^ is mainly regulated by Ca^2+^influx through ligand-gated glutamate receptors, such as N-methyl-D-aspartate receptors (NMDARs) and various voltage-dependent Ca^2+^channels (VDCCs) (Kumar et al., 2009). Moreover, intracellular organelles, such as mitochondria or Endoplasmic Reticulum (ER), and Ca^2+^-binding proteins (CBP) also manage homeostatic mechanisms (Chandran et al., 2019). Intracellular organelles store Ca^2+^, and CBP functions as a buffering agent that reduces the peak intracellular calcium ion concentration (Chandran et al., 2019).

N-methyl-D-aspartate receptors (NMDARs), which are ionotropic non-selective cationic glutamate receptors, play a major role in the rapid control of synaptic plasticity (Kumar et al., 2009). NMDARs are composed of ubiquitously expressed essential subunits (NR1) and modulatory subunits (NR2A-NR2D) (Cull-Candy and Leszkiewicz, 2004). NMDAR activation requires glutamate (binding of ligand), membrane depolarization (to eliminate the Mg^2+^ that blocks the channel), and binding of glycine, which is a co-agonist (Kumar et al., 2009). Although NMDARs are non-selective, they are the most permeable to Ca^2+^ ion (Xin et al., 2005). NMDAR functions within the brain region decline with aging, including learning and memory (Gribkova and Gillette, 2021). There are several reasons why a decrease in NMDAR function due to aging may occur (Kumar et al., 2009). One of the mechanisms is a decrease in the level of NMDAR protein expression, especially its subunits, in the hippocampus with aging (Zhao et al., 2009). The decrease primarily occurs in the CA1 region (Kumar et al., 2009). Specifically, the expression of NR1 and NR2 (NR2A, NR2B) subunit proteins and their mRNA levels have been found to decrease in the aged hippocampus (Kumar et al., 2009). The alteration in the expression of specific NR2 subunits may severely affect NMDAR function in relation to the regulation of the average channel open time and conductance of NMDARs. Modified NR2 subunits induce changes in the time course and magnitude of Ca^2+^ signaling, resulting in reduced Ca^2+^ influx (Cull-Candy et al., 2001). Therefore, a shift in NR2A and NR2B expression triggers developmental changes in cognition and synaptic function (Kumar et al., 2009). Moreover, the NMDAR binding affinity of glutamate decreases during aging and is related to memory decline (Magnusson, 2012). There are several changes in the binding affinity of NMDAR, including glycine or other antagonist (Kumar et al., 2009). Additionally, post-translational modifications of receptors are related to NMDAR functions (Kumar et al., 2009). For example, the phosphorylation state of NMDAR, which is triggered by several kinases such as tyrosine kinase, protein kinase C, and protein kinase A, increases NMDAR-mediated current (Kumar et al., 2009). In contrast, protein phosphatases such as calcineurin act vice versa (Shi et al., 2000). Aging is associated with a disturbance of kinase/phosphatase activity, which is inclined to phosphatase activity, which decreases NMDAR function (Kumar et al., 2009). Therefore, NMDAR function is disturbed by aging due to altered phosphorylation (Kumar et al., 2009). In terms of VDCC, VDCCs are ion channels in the plasma membrane and open during membrane depolarization (Tanaka and Ando, 2001). VDCC enables cellular Ca^2+^influx from the extracellular space. VDCC is classified into L-, P/Q-, and N type channels (Tanaka and Ando, 2001). Interestingly, during aging, L-channels increase in the hippocampus, which inhibits the blockade of L-type VDCCs (Foster, 2007). Increased L-channels lead to an increase in after-hyperpolarization (AFP) in accordance with an increase in Ca^2+^influx (Foster, 2007). Considering that long-term potentiation (LTP) occurs from depolarization and LTP requires blocking specific Ca^2+^ sources, an aging-related increase in the L-channel impairs the LTP threshold and LTP is inhibited (Foster, 2007). Ca^2+^ channels suggest a point of crosstalk between age-related Ca^2+^ regulatory disorders and signaling in neurodegenerative diseases (Foster, 2007).

Intracellular Ca^2+^ reserves include cellular organelles and CBP (Kumar et al., 2009). Cellular organelles, mitochondria, ER, and lysosomes have Ca^2+^ buffering systems, which release and sequester Ca^2+^ (Kumar et al., 2009). In the case of mitochondria, mitochondrial Ca^2+^ induce the TCA cycle to generate energy by neutralizing the polarized negative membrane potential, which is produced from ATP production in the mitochondrial matrix (Chandran et al., 2019). However, aged mitochondria have structural changes in the mitochondrial DNA and mitochondrial membrane, which results in a net reduction in the Ca^2+^ buffering capacity (Kumar et al., 2009). Decreased Ca^2+^ uptake capacity directly contributes to decreased electrochemical gradients across the mitochondrial membrane (Xiong et al., 2004). This mitochondrial depolarization may increase the threshold level of Ca^2+^ required to initiate mitochondrial uptake (Xiong et al., 2004). Consequently, this triggers an age-dependent delay in Ca^2+^ sequestration or the recovery of intracellular Ca^2+^ (Xiong et al., 2004). However, from the perspective of ER, ER regulates Ca^2+^ in two pathways: the inositol (1,4,5)-triphosphate (IP_3_) pathway activated by G protein-coupled receptors (GPCRs) and Ca^2+^induced Ca^2+^release (CICR) (Thillaiappan et al., 2019). In short, the GPCR and CICR pathways regulate Ca^2+^ through IP_3_R and ryanodine receptors (RyRs), respectively (Kumar et al., 2009). However, with aging, the effect of Ca^2+^ dysregulation due to activation of these receptors is cell-specific and depends on other Ca^2+^cellular regulating mechanism (Kumar et al., 2009). For example, CICR is reduced during aging in peripheral synapses because of decreased expression of RyRs, and the reduction in Ca^2+^ amplification in basal forebrain neurons is compensated by mitochondrial buffering (Kumar et al., 2009). Furthermore, recent studies have proposed the possibility of lysosomes acting as Ca^2+^ storage organelles (McGuinness et al., 2007). Although altered lysosomal control of Ca^2+^ is due to aging remains unclear, it has been revealed that the aging brain shows increased lysosomal markers and decreased lysosomal function (Lynch and Bi, 2003). CBP, such as parvalbumin, calbindin-D28K, calretinin, calmodulin, and hippocalcin, which handle the intracellular Ca^2+^ level by rapid Ca^2+^ buffering in cytosol, is regarded to be neuroprotective (Gattoni and Bernocchi, 2019). Generally, with aging, changes include a decrease in the expression of CBP, which is related to the loss of function, and these changes are cell and region specific (Kumar et al., 2009). A decrease in Ca^2+^ buffering or delayed elimination could cause larger or prolonged Ca^2+^responses, which are common in aged neurons (Kumar et al., 2009). Slight alterations in the concentration of Ca^2+^ are fatal for neurons (Kostyuk and Verkhratsky, 1994). However, Ca^2+^ homeostasis in neurons is disturbed by changes in physiological and molecular levels in accordance with increasing age, finally provoking neurodegeneration (Kostyuk and Verkhratsky, 1994).

## Conclusion

Aging is a risk factor for a number of neurodegenerative diseases. There are mechanisms in the normal brain aging process that can converge to neuronal disorders. Under conditions of normal brain aging, the changes were reviewed in terms of structural, biochemical, metabolic, cellular, and molecular mechanisms.

Both gross and microscopic structural changes have been reported. Gross changes include multiple morphological changes, including cerebral atrophy, sulci widening, and cerebrovascular changes. In contrast, microscopically, lipofuscins are accumulated, occupying a large space of cytoplasm where glycoproteins, lipoproteins, and neurotransmitters are synthesized. However, from another perspective, lipofuscin has a positive role in energy production when synergized with myoglobin and respiratory enzymes. Other microscopic changes are associated with amyloid-beta and neurofibrillary tangles, which are regarded as neurotoxic proteins. These changes starve the neurons and have the potential to cause cognition loss. In addition, morphological changes in neuronal cells can be considered aging-related degeneration. Overall, structural changes initiate the destruction of the surrounding neuropil, leading to cognitive damage in dementia. Histopathologic changes appear, especially in the limbic system and most of the CNS.

Regarding the biochemical and metabolic changes involved in aging, several types of neurotransmission molecules are involved, such as acetylcholine, monoamine, and neurosteroid. They affect the intracellular condition by diminishing related enzymes, receptors, and protein functions and triggering an imbalance of neurotransmitters. This disrupted balance influences various regions of the brain, such as the hypothalamus, pituitary gland, and pineal gland, and cascades of dysfunction occur.

Lastly, cellular and molecular changes with aging largely occur in the nucleus, mitochondria, oxidatively managed molecules, lysosomes, proteasomes, electrophysiological regulation and Ca^2+^ homeostasis. During normal brain aging, DNA expression changes, and telomere length are shortened in the nucleus (Figure 1). Conversely, mitochondrial dysfunction is largely caused by mtDNA, ROS, and calcium alterations. These changes have been attributed to abnormal mtDNA integrity, ATP production, and apoptosis. Oxidatively damaged molecules have been reported to be promoted mainly by the respiration of mitochondria. This alteration affects the mitochondrial respiratory system itself, especially in complexes I and III. In addition, these damaged molecules, such as lipids, proteins, and DNA/RNA, along with successive damage, form a vicious cycle that triggers neurodegenerative disorders. As neurons have exotic morphology and function compared with other cells, efficient utilization of energy systems is essential. Lysosomal autophagy and ubiquitin-proteasome functions are important for the reutilization of useful molecules and the removal of cellular waste. However, during the aging process, the functions of the clearance system decrease, and abnormal proteins accumulate. This disturbance contributes to age-related neurodegenerative disorders such as AD and PD. Electrophysiological regulation is controlled by the homeostasis of electrolytes. During senescence, however, the regulation is disturbed by the alteration of channel expression and the modification of the conductive rate of currents. In respect of Ca^2+^ homeostasis, Ca^2+^ alteration is fatal to neurons, and its regulation is tightly regulated through NMDAR, VDCC, mitochondria, ER, and Ca^2+^related proteins. However, due to aging, homeostasis is attributed to alterations in ion exchange systems, a decline in metabolic events, and Ca^2+^related proteins such as receptors. They are also associated with abnormal neuronal functions.

Therefore, normal brain aging entails neurodegenerative changes in the brain. It is worth concentrating on how and why normal aging processes are correlated with neurodegenerative disorders. Through this analysis, a profound understanding of aging in the normal brain and the related factors that generate age-induced neuronal disorders is expected. As everyone ages, it is important to comprehend and limit risk factors to promote healthy brains and quality of life. The mechanism of normal aging-induced neuronal disorders should be considered in the future.

## Author Contributions

H-JK: study concept and design and critical revision of the manuscript. H-JK and JL: acquisition, analysis, and interpretation of data and drafting of the manuscript. Both authors contributed to the article and approved the submitted version.

## Conflict of Interest

The authors declare that the research was conducted in the absence of any commercial or financial relationships that could be construed as a potential conflict of interest.

## Publisher’s Note

All claims expressed in this article are solely those of the authors and do not necessarily represent those of their affiliated organizations, or those of the publisher, the editors and the reviewers. Any product that may be evaluated in this article, or claim that may be made by its manufacturer, is not guaranteed or endorsed by the publisher.

## Abbreviations

GM: gray matter
WM: white matter
AD: Alzheimer’s diseases
PHF: paired helical filament
CNS: central nervous system
MCI: mild cognitive impairment
nAChR: nicotinic acetylcholine receptor
BBB: Brain-blood barrier
AP: action potential
PET: positron emission tomography
NPC: neuro progenitor cell
ROS: reactive oxygen species
mtDNA: mitochondrial DNA
OXPHOS: oxidative phosphorylation
TCA cylce: tricarboxylic acid cycle
ETC: electron transport complex
UPS: ubiquitin-proteasome system
LPO: lipid peroxidation
PD: Parkinson’s disease
PINK1: PTEN-induced novel kinase 1
PSD: postsynaptic density
ER: Endoplasmic Reticulum
NMDAR: N-methyl -D-aspartate receptor
VDCC: voltage-dependent Ca^2+^channels
IP_3_: inositol (1,4,5)-triphosphate
GPCR: G protein-coupled receptors
LTP: long term potentiation
CICR: Ca^2+^induced Ca^2+^ release
RyRs: ryanodine receptors
CBP: Ca^2+^ binding protein.
